# Tumour Tissue Microenvironment Can Inhibit Dendritic Cell Maturation in Colorectal Cancer

**DOI:** 10.1371/journal.pone.0027944

**Published:** 2011-11-18

**Authors:** Adriana J. Michielsen, Andrew E. Hogan, Joseph Marry, Miriam Tosetto, Fionnuala Cox, John M. Hyland, Kieran D. Sheahan, Diarmuid P. O'Donoghue, Hugh E. Mulcahy, Elizabeth J. Ryan, Jacintha N. O'Sullivan

**Affiliations:** 1 Centre for Colorectal Disease, St. Vincent's University Hospital, Elm Park, Dublin, Ireland; 2 Obesity Group, St. Vincent's University Hospital, Elm Park, Dublin, Ireland; 3 School of Biochemistry and Immunology, Trinity College Dublin, Dublin, Ireland; 4 Institute of Molecular Medicine, Trinity Centre for Health Sciences, St James Hospital, Dublin, Ireland; University of Palermo, Italy

## Abstract

Inflammatory mediators in the tumour microenvironment promote tumour growth, vascular development and enable evasion of anti-tumour immune responses, by disabling infiltrating dendritic cells. However, the constituents of the tumour microenvironment that directly influence dendritic cell maturation and function are not well characterised. Our aim was to identify tumour-associated inflammatory mediators which influence the function of dendritic cells. Tumour conditioned media obtained from cultured colorectal tumour explant tissue contained high levels of the chemokines CCL2, CXCL1, CXCL5 in addition to VEGF. Pre-treatment of monocyte derived dendritic cells with this tumour conditioned media inhibited the up-regulation of CD86, CD83, CD54 and HLA-DR in response to LPS, enhancing IL-10 while reducing IL-12p70 secretion. We examined if specific individual components of the tumour conditioned media (CCL2, CXCL1, CXCL5) could modulate dendritic cell maturation or cytokine secretion in response to LPS. VEGF was also assessed as it has a suppressive effect on dendritic cell maturation. Pre-treatment of immature dendritic cells with VEGF inhibited LPS induced upregulation of CD80 and CD54, while CXCL1 inhibited HLA-DR. Interestingly, treatment of dendritic cells with CCL2, CXCL1, CXCL5 or VEGF significantly suppressed their ability to secrete IL-12p70 in response to LPS. In addition, dendritic cells treated with a combination of CXCL1 and VEGF secreted less IL-12p70 in response to LPS compared to pre-treatment with either cytokine alone. In conclusion, tumour conditioned media strongly influences dendritic cell maturation and function.

## Introduction

Dendritic cells (DCs) are potent antigen presenting cells capable of activating naïve T cells. DCs are present in tissues in an immature state and display low levels of maturation or co-stimulatory markers such as CD83, CD80 or CD86. Immature DCs (iDCs) recognise and capture specific antigens, including tumour antigens. DCs undergo a functional maturation process in response to inflammatory mediators such as IFN-α or Toll like receptor (TLR) agonists. As DCs mature they gain the potential of presenting antigen to T cells and activating a specific anti-tumour T cell response [1], [2]. DCs that secrete high levels of bioactive IL-12p70 induce optimal anti-tumour immunity, as they have increased capacity to enhance natural killer cell activity, skew the response to Th1 and prime tumour specific CD8^+^ T cells [3], [4].

However, many tumours evade the immune response by secreting cytokines and other factors that inhibit DC differentiation or the maturation of tumour infiltrating DCs. [1]. One of these ‘pro-tumour’ factors, Vascular Endothelial Growth Factor (VEGF) is known for sustaining tumour growth via its angiogenic properties but can also elicit an inhibitory effect on DC differentiation and maturation, enhancing tumour survival [1], [5], [6], [7], [8]. VEGF has successfully been targeted by the humanised monoclonal antibody Bevacizumab (Avastin) [9], however response rates are approximately 40% and many patients develop resistance to this treatment. Therefore, it is crucial to explore the potential of other inflammatory mediators present in the tumour microenvironment that may inhibit DC maturation, as these may also be potential therapeutic targets.

Several cytokines and chemokines are present at high levels in the tumour microenvironment, compared to normal tissues, such as CCL2 (MCP-1), CXCL1 (GROα) and CXCL5 (ENA-78) [10], [11], [12]. CCL2 is known to attract monocytes, T-cells and dendritic cells [10], [13], while the main function of CXCL1 and CXCL5 is to attract and activate neutrophils [14], [15]. In addition to their chemoattractant functions, CCL2, CXCL1 and CXCL5 also play an important role in angiogenesis [15], [16], [17], demonstrating the multifunctional nature of these chemokines. It is known that human myeloid DCs express CCR2 and CXCR2, the receptors for CCL2 and CXCL1 and CXCL5, respectively [18], [19]. However the effect of these chemokines on DC maturation and function has not previously been investigated.

In this study, we used explanted human colorectal cancer tissue to model the tumour microenvironment [20]. Explant tissues maintain the complex 3D structure of the tumour, including the stroma, thus allowing the production of many different tumour associated factors, closely mimicking the inflammatory milieu of the tumour *in situ*. Previous studies have shown that supernatants from tumour cell lines can inhibit DC maturation [21], [22], however the importance of factors secreted by the entire tumour on dendritic cell maturation has not been previously examined.

We demonstrate that Tumour Conditioned Media (TCM) from cultured colorectal cancer tumours significantly inhibited LPS induced DC maturation, markedly increasing IL-10 while decreasing IL-12p70 secretion in response to LPS. We found that the TCM contained significant amounts of the chemokines CCL2, CXCL1 and CXCL5 in addition to VEGF. Levels of CCL2, CXCL1 and CXCL5 in the TCM correlated with CD83 expression and IL-12p70 secretion from DCs treated with TCM. Individually, all of these inflammatory mediators significantly inhibited LPS induced IL-12p70 secretion, however IL-10 secretion remained unaffected. Interestingly, the effects of CXCL1 and VEGF on the inhibition of LPS induced IL-12p70 secretion by DCs were additive. In addition, CXCL1 also had a marked inhibitory effect on LPS induced up-regulation of HLA-DR.

## Results

### Tumour conditioned media inhibits DC maturation and IL-12p70 secretion while augmenting IL-10 secretion in response to LPS

**Figure 1:**
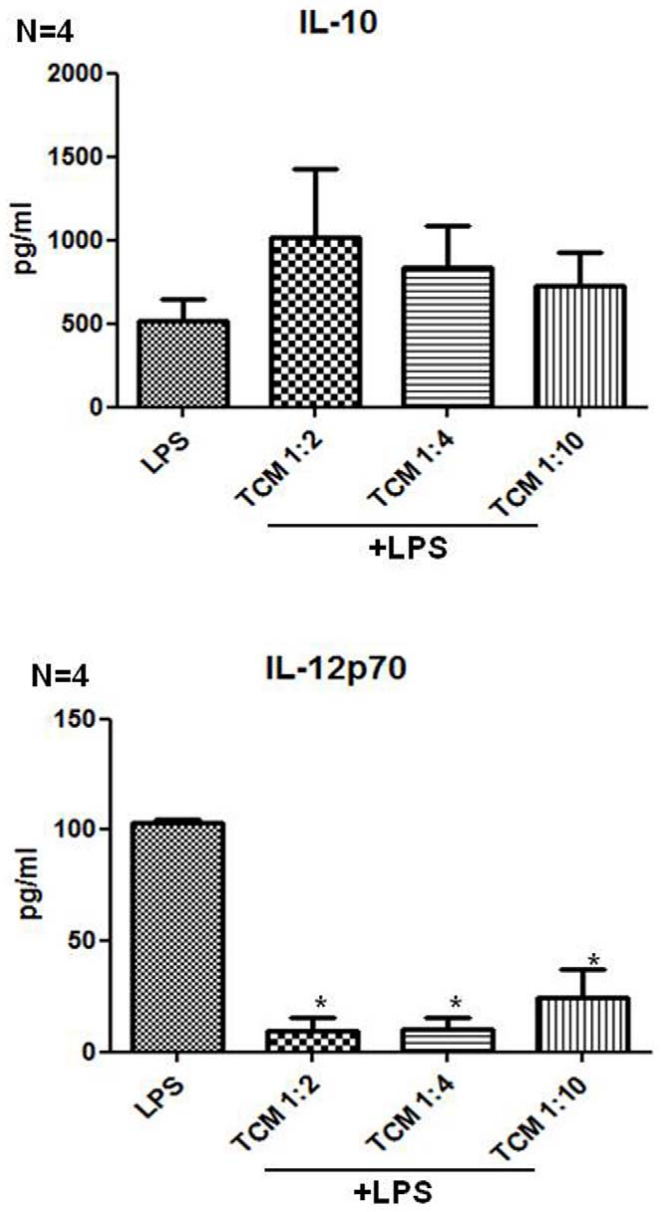
Tumour Conditioned Media affects IL-10 and IL-12p70 secretion in a dose dependent manner. Immature monocyte derived dendritic cells (iDCs) (n = 2) were treated for 4 hours with 1∶2, 1∶4 and 1∶10 dilutions of Tumour Conditioned Media (TCM) obtained by culturing the explanted tumours of 4 colorectal cancer patients *in vitro* for 72 hours. LPS (1 µg/mL) was added and the cells were cultured for a further 18 hours. Supernatants were collected from the cells cultured as described above, and the levels of IL-10 and IL-12p70 secretion by the DCs were measured by ELISA. Statistical differences were determined by Mann-Whitney U test.

Monocyte derived DCs obtained from 2 healthy volunteers were incubated with different volumes of TCM from 4 colorectal cancer patients for 4 hours prior to LPS simulation for a further 18 hours, to determine the optimal concentration of TCM. We examined the effect of TCM on the ability of DCs to secrete IL-10 and IL-12p70 in response to stimulation by LPS. Secretion of IL-12p70 by DCs augments and directs the expansion and differentiation of tumour specific Th1 responses while high levels of IL-10 secretion preferentially leads to the expansion of immunosuppressive T regulatory cells. Treatment of DCs with a 1 in 2 dilution of TCM showed to have the most potent effect on IL-10 increase and IL-12p70 inhibition (p = 0.028) in response to LPS (Figure 1). No significant difference was observed in expression of CD80, CD86, CD54, CD83 and HLA-DR (MHC II), markers associated with DC maturation, between different dilutions of TCM (data not shown). Therefore we used a 1 in 2 dilution of TCM for further analysis.

**Figure 2:**
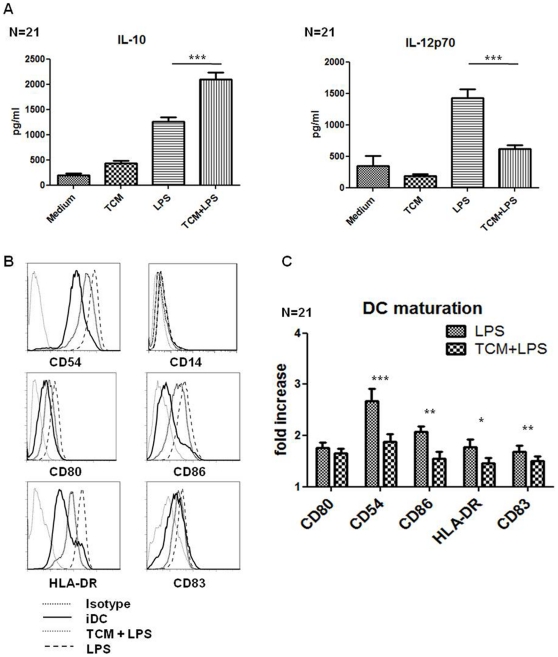
Tumour Conditioned Media inhibits dendritic cell maturation, augments IL-10 while inhibiting IL-12p70 secretion induced by LPS. iDC (n = 6) were treated with TCM of 21 colorectal cancer patients for 4 hours before adding LPS; the cells were cultured for a further 18 hours. Levels of IL-10 and IL-12p70 secretion by the DCs were measured by ELISA. Statistical differences were determined by ANOVA. (A) Expression of the following maturation markers: CD80, CD54, CD86, HLA-DR, and CD83 were assessed by flow cytometry. One representative experiment of six is shown (B). The Fold Change in M.F.I. of each marker was calculated relative to unstimulated iDCs for cells treated with LPS or TCM+LPS. Statistical significance was calculated using the Wilcoxon signed rank test. (C). * *p<*0.05, ** *p*<0.01, and *** *p*<0.001.

Monocyte derived DCs obtained from 6 healthy donors were treated with 1 in 2 TCM of 21 colorectal patients for 4 hours before treatment with LPS for a further 18 hours, and interestingly, TCM treated DCs secreted significantly higher levels of IL-10 in response to LPS (p<0.0001), but significantly lower levels of IL-12p70 (p<0.0001) (Figure 2A). In addition IL-12p70 showed a negative correlation with CD83 expression Spearman r = −0.71, p<0.001 (data not shown), further suggesting that TCM treated DCs with increasing CD83 expression have decreasing levels of IL-12p70 secretion.

**Figure 3:**
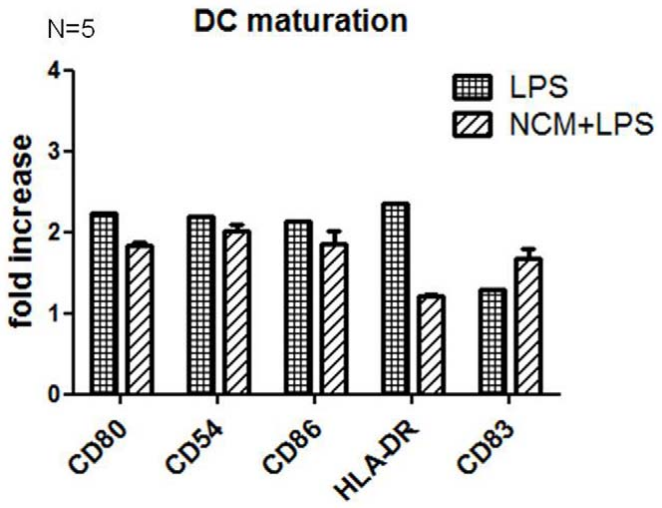
Normal Conditioned media does not affect LPS induced DC maturation. Normal conditioned media (NCM) of 5 colorectal cancer patients was used to treat monocyte derived DCs before adding LPS. Expression of CD80, CD54, CD86, HLA-DR and CD83 was measured by flow cytometry. No statistical significant difference was observed. Statistical analysis was performed using the Wilcoxon signed rank test.

Treatment of DCs with TCM also significantly inhibited LPS-induced DC maturation, with reduced expression of CD54 (*p*<0.0001), CD86 (*p* = 0.0035), HLA-DR (*p* = 0.0474) and CD83 (*p* = 0.0018) observed (Figure 2B & C). Treatment of DCs with conditioned media taken from normal tissue adjacent to the tumour tissue (NCM) did not inhibit LPS induced maturation or cytokine secretion from DCs (Figure 3).

### Levels of CCL2, CXCL1, and CXCL5 present in TCM correlate with CD83 expression and IL-12p70 secretion from DCs

**Figure 4:**
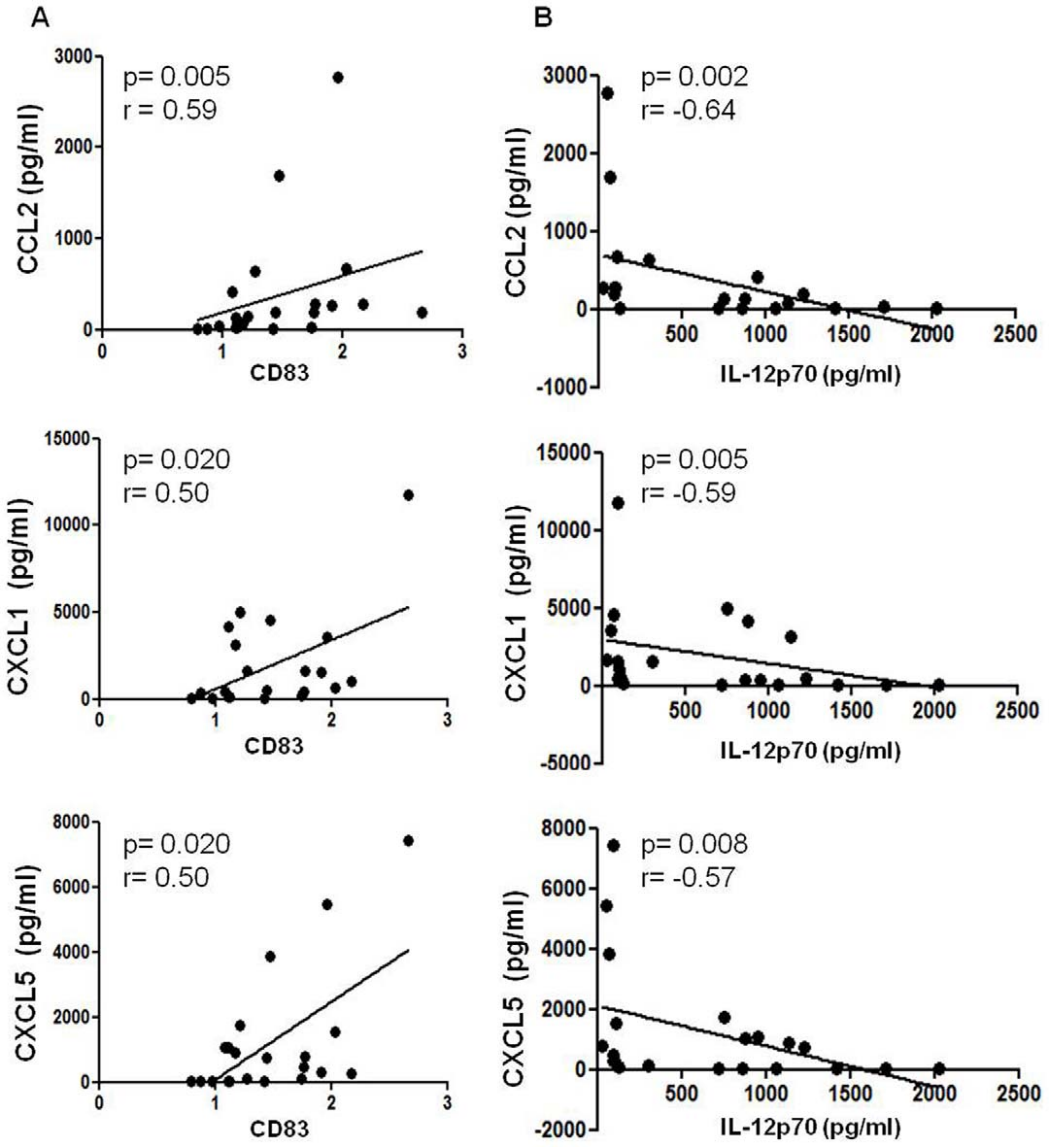
Levels of CCL2, CXCL1, and CXCL5 in TCM correlate with CD83 expression and IL-12p70 secretion. Concentrations of CCL2, CXCL1, CXCL5 and VEGF in tumour conditioned media of 21 colorectal cancer patients were measured by ELISA. The concentrations of CCL2, CXCL1 and CXCL5 correlated with expression of CD83 on DCs treated with TCM (A). CCL2, CXCL1 and CXCL5 inversely correlated with secretion of IL-12p70 from dendritic cells treated with the TCM (B). The Spearman rank correlation test was used.

TCM obtained from 21 patients with colorectal cancer were analysed by ELISA for several known tumour associated factors. Levels of CCL2, CXCL1 and CXCL5 were consistently higher than those of VEGF (a current therapeutic target in colorectal cancer) in all patients examined. We found that levels of CCL2 (Spearman r = 0.59, *p* = 0.0053) CXCL1 (Spearman r = 0.50, *p* = 0.0202) and CXCL5 (Spearman r = 0.50, *p* = 0.0199) present in the TCM positively correlated with CD83 expression on DCs treated with TCM (Figure 4A). This implies that with increasing levels of CCL2, CXCL1 and CXCL5 present in the TCM, CD83 expression is elevated on DCs treated with TCM. Even though VEGF is present at detectable levels in the TCM, it showed no correlation with DC maturation. In addition we found that IL-12p70 secretion from DCs treated with TCM negatively correlated with CCL2 (Spearman r = −0.64, *p* = 0.0019), CXCL1 (Spearman r = −0.59, *p* = 0.0049) and CXCL5 (Spearman r = −0.57, *p* = 0.0075) (Figure 4B). This suggests that with increasing levels of CCL2, CCL1 and CXCL5 in the TCM, IL-12p70 secretion from DCs decreases. There was no significant correlation between the four cytokines and the other DC markers, CD80, CD54, CD86, and HLA-DR, and IL-10 secretion.

### Effect of CCL2, CXCL1, CXCL5 and VEGF on DC maturation marker expression and cytokine secretion in response to LPS

**Figure 5:**
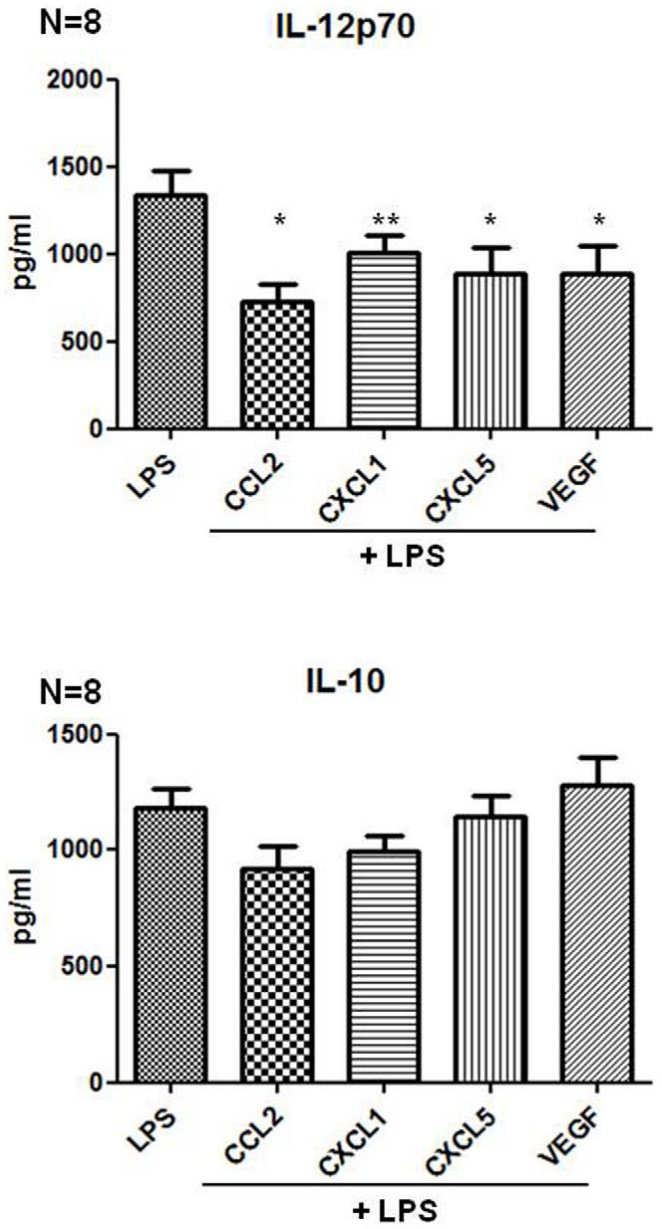
CCL2, CXCL1, CXCL5 and VEGF inhibit IL-12p70 secretion by DCs in response to LPS. The supernatants of the DCs cultured in the presence of CCL2, CXCL1, CXCL5 and VEGF (n = 8) were harvested and concentrations of IL-10 and IL-12p70 determined by ELISA. Statistical significance was determined by the Mann-Whitney U test. * p<0.05, ** p<0.01.

Having established that TCM inhibits DC maturation in response to LPS and that TCM contains high levels of the chemokines CCL2, CXCL1 and CXCL5 we next determined if these individual components had a direct effect on DCs. Recombinant VEGF significantly inhibited LPS-induced expression of DC maturation markers CD80 (*p* = 0.0391) and CD54 (*p* = 0.0078) and CXCL1 significantly inhibited the upregulation of HLA-DR (*p* = 0.0156) (Data not shown). Although CCL2 and CXCL5 showed a reduction of LPS-induced maturation of DCs, this did not reach statistical significance (Data not shown). While the pre-treatment of DCs with CCL2, CXCL1, CXCL5 or VEGF failed to augment LPS-induced IL-10 secretion, a significant decrease in the levels of LPS-induced IL-12p70 secretion was observed: CCL2 (*p* = 0.048), CXCL1 (*p* = 0.0068), CXCL5 (*p* = 0.0255), VEGF (*p* = 0.0402) (Figure 5). IL-1β, TNF-α, IL-8 and IL-6 production by DCs in response to LPS in the absence or presence of pre-treatment with VEGF, CCL2, CXCL1 or CXCL5 were also measured; however there were no significant differences in the levels of these cytokines determined under these conditions (data not shown). Treatment of DCs with CCL2, CXCL1, CXCL5 and VEGF did not affect their ability to induce T cell proliferation or production of IFNγ (data not shown).

In addition we investigated whether CCL2, CXCL1, CXCL5 and VEGF could affect DC migration. CCL19 was used as a positive control since it is known to bind to the chemokines receptor CCR7, which is expressed by DCs [23]. We found that they did not increase migration compared to CCL19 (data not shown).

### CXCL1 and VEGF have an additive inhibitory effect on IL-12p70 secretion by DCs in response to LPS

**Figure 6:**
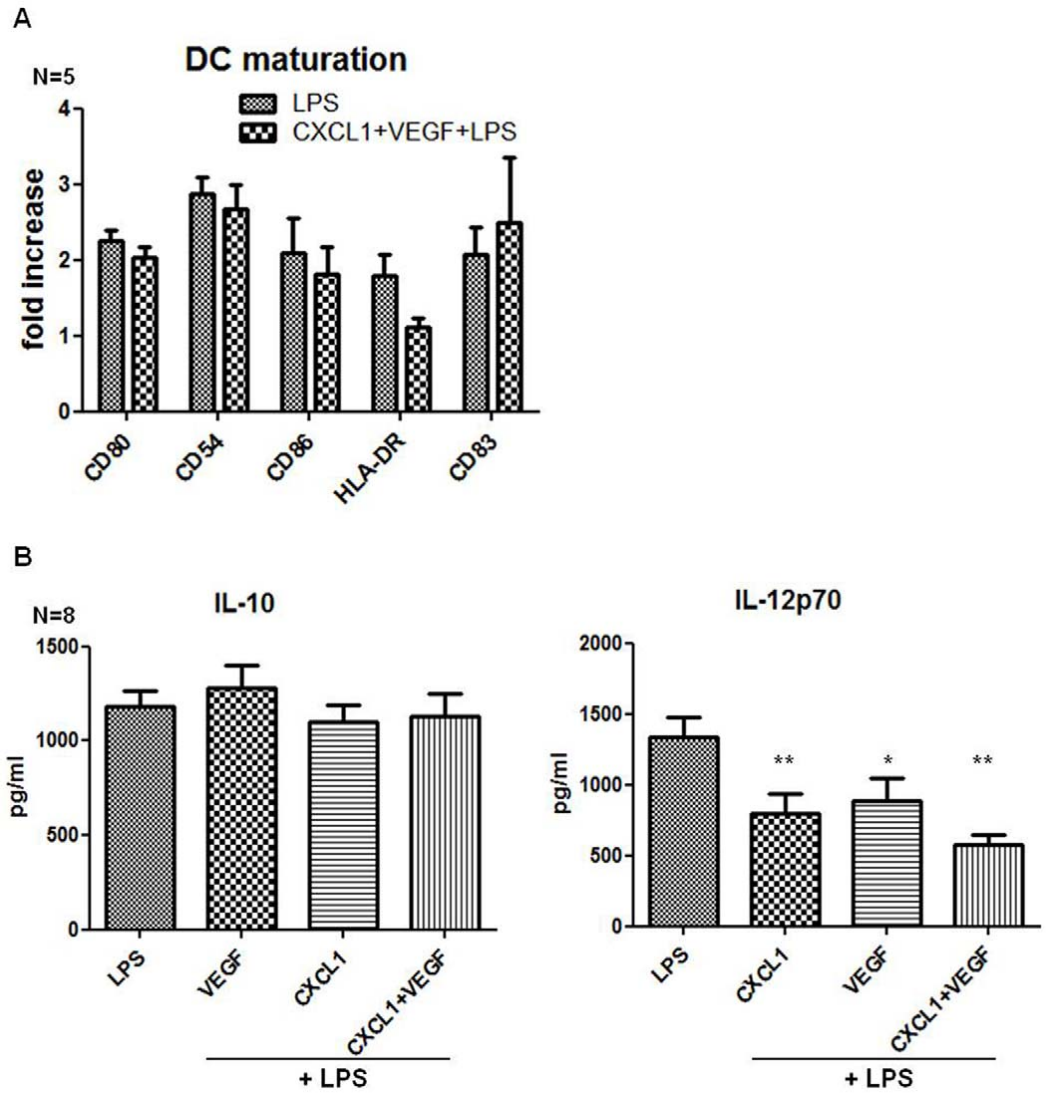
The combination of CXCL1 and VEGF inhibits LPS induced IL-12p70 secretion by DCs. iDCs (n = 5) were treated with a combination of recombinant CXCL1 (100ng/mL) and VEGF (20 ng/mL), 4 hours prior to the addition of LPS (1 µg/mL). The cells were then cultured for a further 18 hours and the levels of CD80, CD54, CD86, HLA-DR and CD83 were assessed by flow cytometry. Results are shown as fold-change in M.F.I. relative to untreated iDCs. Statistical significance was determined by Wilcoxon signed rank test (A). Supernatants were removed from the cells treated as described above and the concentration of IL-10 and IL-12p70 was determined by ELISA. Statistical significance was calculated by Mann-Whitney U test (B). * p<0.05, ** p<0.01.

The TCM has a potent inhibitory effect on DC maturation and function, but CCL2 and CXCL5, which are present at high levels in TCM, do not have a significant effect on dendritic cell maturation marker expression, while CXCL1 only significantly inhibited HLA-DR and VEGF significantly inhibited CD80 and CD54 (data not shown). Therefore, we investigated whether combining the recombinant cytokines might have a more potent effect on dendritic cell maturation. We examined seven different combinations of the four cytokines: CCL2+CXCL1, CCL2+CXCL5, CCL2+VEGF, CXCL1+CXCL5, CXCL1+VEGF, CXCL5+VEGF, CCL2+CXCL1+CXCL5+VEGF. The combination of CXCL1 and VEGF did not significantly inhibit LPS-induced maturation marker expression by DCs (CD80 *p* = 0.0625, CD54 *p* = 0.6, CD86 *p* = 0.6, HLA-DR *p* = 0.1, CD83 *p* = 0.8) (Figure 6A). However combining CXCL1 and VEGF significantly reduced LPS-induced IL-12p70 (*p* = 0.0028), but did not alter the levels of IL-10 (Figure 6B). IL-1β, TNFα, IL-8 or IL-6 secretions were not altered (data not shown). No other combination had a direct effect on LPS-induced DC maturation or cytokine secretion of DCs in response to LPS when compared to the individual cytokines (data not shown). In addition inclusion of neutralising antibodies to CXCL1 and VEGF, alone and in combination, did not correct TCM induced alteration of DC maturation, IL-10 or IL-12p70 secretion from DCs in response to LPS (data not shown).

## Discussion

In this study we have shown for the first time that tumour conditioned media from colorectal cancer tumour explants can significantly inhibit LPS-induced maturation of monocyte derived DCs. In addition, treatment of DCs with TCM prior to LPS stimulation significantly increased production of IL-10, an anti-inflammatory cytokine that inhibits a Th1 response [24], while decreasing the secretion of IL-12p70, a pro-inflammatory cytokine required for Th1 responses: a DC phenotype associated with tolerance. Subsequently, we found that CCL2, CXCL1 and CXCL5 were expressed at high levels in the TCM compared to VEGF, and we determined if these inflammatory mediators could alter the function of DCs. VEGF was included in our analyses as it had previously been shown to inhibit DC maturation [8], [22]. The addition of recombinant VEGF to iDCs had an inhibitory effect on the LPS-induced upregulation of CD80 and CD54, while adding recombinant CXCL1 to iDCs decreased HLA-DR expression following LPS treatment.

However, treatment of iDCs with human recombinant CCL2, CXCL1, CXCL5 or VEGF, at doses in line with other studies [13], [22], [25], [26], resulted in significantly reduced levels of IL-12p70 secretion in response to LPS stimulation. These data suggest that inflammatory mediators present in the tumour microenvironment reduce the capacity of local DCs to secrete IL-12p70 and thus the induction of an effective anti-tumour response.

Similar findings were reported in a study investigating the effect of conditioned media taken from the pancreatic cancer cell line BxPC-3 (BxCM), where DC differentiation and maturation was inhibited by BxCM. The study showed that BxCM reduced the expression of CD83, CD1a, CD1c, CD80 and CD86 by DCs in response to TNFα. Consistent with our findings, this study found a significant increase in IL-10 and a reduction in IL-12p70 production in response to stimulation with TNFα following BxCM treatment [27]. However, the treatment of DCs with supernatant of RCC-10 (renal cell carcinoma cells), resulted in a reduction in the expression of maturation markers, but did not affect IL-10 production in response to stimulation with TNFα, IFNα and poly I:C [22]. These studies, together with the data presented here, suggest that many tumour types secrete inflammatory mediators that can inhibit the functionality of DCs; but that there is likely to be variation in the inflammatory milieu and the resultant effect on DC function according to tumour type and stage. While it would have been interesting to assess and isolate the actual levels of DC infiltration in the tumour explant tissue, this could not be performed due to the limitation of explant tissue size we received from surgery. Our results indicate that NCM does not significantly affect DC maturation or cytokine secretion, indicating that the normal adjacent tissue may have functional immunity. There was no evidence of an immunosuppressive field effect.

VEGF is established as an important factor contributing to tumour growth by inducing angiogenesis. VEGF is a therapeutic target in several cancers, including colorectal cancer, where the humanised anti-VEGF mAb, Bevacizumab (Avastin) is employed. Previous studies have demonstrated that VEGF inhibits DC maturation and DCs ability to activate T cells [5], [8], [22]. In this study, we demonstrate that VEGF inhibits the expression of CD80 and CD54, but not CD86 or HLA-DR, data consistent with Alfaro *et al.* [22]. Interestingly, we found that VEGF treated DCs secreted significantly reduced levels of IL-12p70 in response to LPS; a finding that has not been previously documented. The use of LPS to mature the DCs, versus the maturation cocktail consisting of TNFα, IFNα and poly I:C used by Alfaro *et al.* might explain the differences observed in the effect of VEGF on DC maturation and IL-12p70 secretion. Our finding that VEGF stimulation of DCs does not affect T cell proliferation and cytokine secretion is consistent with Alfaro *et al.*, however they found an inhibitory effect of VEGF when present at the differentiation stage of DC development, indicating that differentiated DCs might be less responsive to the effects of VEGF [22]. Our study also showed that our colorectal cancer patients explant tissue secreted high levels of CCL2, CXCL1 and CXCL5, compared to VEGF. Interestingly, CCL2, CXCL1 and CXCL5 levels in TCM correlated with CD83 expression on TCM treated DCs. This is the first time this correlation has been observed, and suggests that patients with higher levels of CCL2, CXCL1 or CXCL5 might have increased levels of CD83 on DCs. CD83 is very important for naive T-cell and B cell activation [28], [29], however we also found that enhanced expression of CD83 on DCs treated with TCM correlated with reduced IL-12p70 secretion from TCM treated DCs. It has been previously reported that while different stimuli might mature DCs to similar levels, with similar expression of CD83, CD80 and CD86, the profile of cytokines that they secrete differ; stimuli such as IFN-γ can result in IL-12 secreting CD83+ DCs that stimulate Th1 responses while other stimuli such as prostaglandin E2 result in CD83+ DCs that secrete no IL-12 but efficiently promote a Th2 response [30].

While it has been reported that CCL2, CXCL1 and CXCL5 are important for tumour growth, proliferation, and angiogenesis [31], [32], the effect of these chemokines on DCs has not previously been documented. CCL2, CXCL1 and CXCL5 levels in TCM correlated inversely with IL-12p70 secretion, this is the first time this correlation has been observed, and suggests that with increasing levels of CCL2, CXCL1 and CXCL5 in the TCM, IL-12p70 secretion from DCs is reduced. CCL2 is a chemoattractant for monocytes, memory T-cells and dendritic cells [26], [31]. CCL2 has also been previously reported to induce angiogenesis [17], which is important for tumour growth and metastasis. Both pro- and anti-tumour effects of CCL2 have been observed through its effect on monocytes and macrophages [31], while here we show that CCL2 may also influence the anti-tumour immune response by affecting the ability of DCs to secrete IL-12p70 but not other cytokines including IL-10, IL-1β, IL-6, IL-8 or TNF-α in response to LPS stimulation. In contrast, Braun *et al.* have previously shown that CCL2 can inhibit IL-12p70 production from monocytes and this is pertussis toxin sensitive, but they did not observe this reduction in IL-12p70 secretion from dendritic cells treated with either SAC+IFNγ or CD40L+IFNγ [33]. Alternatively, Omata *et al.* found DCs differentiated from monocytes in the presence of CCL2 had a reduced capacity to secrete IL-12p70 following stimulation with CD40 ligand, an effect not sensitive to pertussis toxin. These authors reported no effect of CCL2 on the differentiation or maturation of DCs. [34]. Therefore, the precise mechanism by which CCL2 exerts its effect on DCs remains to be fully elucidated.

While the effect of CXCL1 and CXCL5 on DC maturation and cytokine secretion has not been previously investigated, their known function is to attract and activate neutrophils [14], [15], which in turn have been implicated with tumour cell growth, angiogenesis and metastasis [35]. Since DCs express CXCR2 [19] which is the receptor for CXCL1 and CXCL5, it is not surprising that CXCL1 and CXCL5 could have an effect on DC function. Chemokines such as CCL2, CXCL1 and CXCL5 signal through G-protein coupled receptors, which control intracellular cAMP levels and increased cAMP levels correlates with decreased IL-12 production [36]. However, the mechanism by which these chemokines reduce IL-12p70 secretion from DCs is not known, and it will be subject of our future investigations.

Interestingly CXCL1 and VEGF have an additive inhibitory effect on IL-12p70 secretion. They act on different receptors which have previously been shown to be present on DCs [7], [19], therefore they might activate different signalling pathways simultaneously, or they could activate the same pathway more effectively. VEGF has been shown to enhance phospho-ERK1 & ERK2 in DCs and this pathway negatively regulates monocyte derived DC maturation [7], [37]. Which signalling pathways are activated by CXCL1 through CXCR2 in dendritic cells is yet unknown, however studies in different cell types, such as cancer cells, show that CXCR2 signalling can activate NF-κB, STAT3 and the ERK pathways [38], [39], [40]. Hence, there are a variety of potential mechanisms by which CXCR2 ligands may inhibit the secretion of IL-12p70 by DCs. However, immune depleting either of these cytokines singly or in combination with neutralising antibodies did not reverse the inhibitory effect of the TCM. This proves that it is probably multiple different factors acting together that cause DC inhibition. This is consistent with previous research showing that blocking of a single factor, VEGF in supernatants of RCC-10 cells did not have a significant effect on DC activation [22].

We observed that CCL2, CXCL1, CXCL5 and VEGF do not block DC maturation, nor do they affect DC migration or T-cell proliferation. Unlike DCs treated with TCM, the individual inflammatory mediators do not enhance IL-10 however they all inhibited IL-12p70 secretion; an effect not previously shown for CCL2, CXCL1, CXCL5 and VEGF. The explant tumour tissues secrete many different soluble factors, and it is not very surprising that a few isolated factors on their own do not have an effect comparable to TCM. It is very likely that the many different factors secreted into the TCM by the explant tumour act together in inhibiting DC maturation and function. However the ability of CCL2, CXCL1, CXCL5 and VEGF to significantly reduce IL-12p70 production by DCs is important, as this may potentially result in a reduced anti-tumour Th1 response *in vivo*.

In conclusion we found that TCM inhibits DC maturation, and induces IL-10 while inhibiting IL-12p70 secretions from DCs. The TCM components CCL2, CXCL1, CXCL5 and VEGF seem to play a role in modulating the inflammatory response through inhibition of IL-12p70 secretion by DCs, possibly to protect the tumour from a potent immunologic response against it. In addition, CXCL1 and VEGF act together in the inhibition of IL-12p70 secretion from DCs. Even though CCL2, CXCL1, CXCL5 and VEGF are present in the TCM at high levels, they are not the main components of the TCM that affect DC maturation and function. In conclusion, we have demonstrated the importance of tumour conditioned media in regulating dendritic cell function in colorectal cancer patients, however the underlying mechanisms still need to be elucidated.

## Materials and Methods

### Ex vivo tumour explant culture

All tissue was obtained with the informed written consent of the patient, and the protocol was approved by the Ethics Committee of St. Vincent's University Hospital.

Surgically resected colorectal cancer tumour tissue (n = 21) and normal tissue (>10cm from the tumour; n = 5) was obtained from patients from the Centre for Colorectal Disease's explant tissue bio-bank at St. Vincent's University Hospital, Dublin (12 male, 9 female, median age 67, 1 Stage II, 5 Stage III, 15 Stage IV colorectal cancer). The explanted tissue was cut into at least 4 equal-sized pieces of approximately 5 mm^3^ and cultured as previously described [20]. Briefly, the explanted tumour tissues were cultured for 72 h (in 24 well plates) in 2 mL RPMI 1640 containing 100 U/mL Penicillin, 100 µg/mL Streptomycin, 4 µg/mL Fungizone, 30 µg/mL gentamicin (Invitrogen; Carlsbad, California) and supplemented with 20% foetal bovine serum (Invitrogen). Following 72 hours in culture, Tumour Conditioned Media (TCM) and normal conditioned media (NCM) was collected and stored at −20°C until used for analyses.

### Dendritic cell isolation and culture

Human monocyte-derived immature dendritic cells (iDCs) were generated from peripheral blood mononuclear cells (PBMCs) obtained from buffy coat preparations (National Blood Centre, St. James Hospital, Dublin). Monocytes were isolated by positive selection using CD14-magnetic beads (Miltenyi Biotec, Bergisch Gladbach, Germany) and seeded at a density of 1×10^6^ cells/mL in 6-well plates in 3 mL of RPMI 1640 containing 100 U/mL Penicillin, 100 µg/mL Streptomycin, 4 µg/mL Fungizone and supplemented with 10% defined Hyclone foetal bovine serum (Thermo Fisher Scientific, Waltham, MA, US), human granulocyte-macrophage colony-stimulating factor (GM-CSF; 50 ng/mL, Immunotools, Friesoythe, Germany), and human IL-4 (70 ng/mL; Immunotools). Cells were fed at day 3 by replacing half the medium and adding fresh cytokines. At day 6 the cells exhibited an immature DC phenotype (CD14^-^, CD11c^+^, CD86^-^, CD54^low^, CD83^-^, CD80^-^, and HLA-DR^low^).

### Stimulation of monocyte derived DC

Freshly isolated iDC were plated in triplicate in 96 well plates at 1×10^5^ cells/200 µl in RPMI 1640 media supplemented with 10% defined Hyclone FBS (Thermo Fisher Scientific), and stimulated with 1 in 2, 1 in 4 and 1 in 10 dilution of Tumour Conditioned Media (TCM) from 4 cultured explant tissues for 4 hours before adding 1 µg/mL *Escherichia coli* lipopolysaccharide (LPS; Alexis Biochemicals, Lausen, Switzerland) to determine the best dose of TCM. Subsequently DCs were treated with a 1 in 2 dilution TCM of all 21 patient explant tissues for 4 hours before adding 1 µg/mL LPS. In addition, DCs were treated with a 1 in 2 dilution of NCM of 5 patient explant tissues for 4 hours before 1 µg/ml LPS was added. In separate experiments 1×10^5^ iDC/200 µl media were treated with human recombinant 50 ng/mL CCL2 (MCP-1), 100 ng/mL CXCL1 (GROα), 100 ng/mL CXCL5 (ENA-78) and 20 ng/mL VEGF (R&D Systems, Abingdon, UK) for 4 hours before 1 µg/mL LPS was added to the samples. Cultures were incubated for a further 18 h at 5% CO_2_ and 37°C. In addition, iDCs were cultured with a combination of cytokines (at the concentrations described above): CCL2+CXCL1, CCL2+CXCL5, CCL2+VEGF, CXCL1+CXCL5, CXCL1+VEGF, CXCL5+VEGF, CCL2+CXCL1+CXCL5+VEGF for 4 hours before LPS was added for a further 18 hours. Supernatants were harvested and levels of IL-10 and IL-12p70 secretion analysed and cells were assessed for expression of maturation markers by flow cytometry as described below.

### Neutralising CXCL1 and VEGF in TCM

iDC were treated with TCM of 4 colorectal cancer patients, as described above, with the addition of neutralising antibodies to VEGF (Avastin,100 µg/mL), CXCL1 (15 µg/ mL, R&D systems) and corresponding isotype control (15 µg/mL, R&D Systems) for 4 hours before LPS was added for 18 hours. Cells were assessed for expression of maturation markers as described below, and levels of IL-10 and IL-12p70 in the supernatant were analysed using ELISA.

### Flow cytometry

Dendritic cells were stained with the following monoclonal antibodies (mAb): fluorescein isothiocyanate (FITC)-conjugated anti-CXCR2 (R&D systems) anti-CD14 and anti-CD80; phycoerythrin (PE)–conjugated anti-CD54; Phycoerythrin-Cy5 (PeCy5)–conjugated anti-CD86 and anti-CD83; allophycocyanin (APC)–conjugated anti-CD11c and anti-HLA-DR (all from BD Biosciences, Oxford, UK). Cells were also stained with corresponding isotype control mAbs (BD Biosciences). Cells were acquired on FACScalibur flow cytometer and the data were analysed with CellQuest Pro (BD Biosciences), or Flowjo software (Tree Star Inc., Ashland, OR).

### Quantification of cytokines by ELISA

Levels of CCL2, CXCL1, CXCL5, and VEGF in TCM; IL-10 and IL-12p70 in DC supernatant; IFNγ in T cell supernatant were quantified by sandwich ELISA according to the manufacturer's protocol (R&D Systems). IL-1β, IL-6, IL-8 and TNF-α in DC supernatants were measured by MSD multiplex assays as per manufacturer's instructions (Meso Scale Discovery, Sector Imager 2400, Gaithersburg, Maryland 20877).

### Dendritic cell migration assay

Monocyte derived dendritic cells were seeded at 5×10^5^ cells/ml in the upper chambers of 96 well plate (Corning B.V. Life Sciences, Amsterdam, The Netherlands). CCL2, CXCL1, CXCL5, VEGF and CCL19 (Immunotools) were added to the lower chambers at 50 ng/ml and 150 ng/ml (n = 3). Plates were incubated for 4 hours at 37°C. Cells were counted using a Z1 Coulter Particle counter (Beckman Coulter Diagnostics Limited, Lismeehan, O'Callahans Mills, Ireland).

### T cell proliferation assay

CD3^+^ T cells were isolated from PBMCs of healthy donors using CD3-labelled magnetic beads (Miltenyi Biotec). The T cells were labelled with CFSE (Molecular Probes, Eugene, OR). Briefly, cells were incubated with 0.5 µM CFSE in PBS for 2.5 min. Heat inactivated FBS (Invitrogen) was added to stop the reaction and cells were washed prior to resuspension in complete RPMI. T cells were incubated with DCs in round-bottomed 96-well plates for 5 days in a 10∶1 ratio (T cells:DCs). Cells were harvested for flow cytometric analysis and levels of IFNγ in the supernatant were determined by ELISA.

### Intracellular IFNγ

PBMC's form healthy donors were incubated with DCs in a 10∶1 ratio (PBMC: DCs) for 5 days. Cells were harvested and treated for intracellular staining of IFNγ as follows: Brefeldin A (10 µg/ml, Sigma-Aldrich, Dublin, Ireland) phorbol myristate acetate (PMA) (25 ng/ml, Sigma-Aldrich) and Ionomycin (1 µg/ml, Sigma-Aldrich) was added to the cells for 4 hours to stimulate cytokine production and prevent secretion of these cytokines. The cells were stained with CD3-APC (BD biosciences) before fixing the cells with 4% paraformaldehyde (Sigma-Aldrich) for 10 minutes and adding 0.2% w/v saponin (Sigma-Aldrich) for another 10 minutes to permeabilise the cell membrane. 5 µl of PE conjugated anti-IFNγ (BD Biosciences) was added and cells were analysed by flow cytometry.

### Statistical Analysis

Statistical analyses were performed using GraphPad Prism version 5.00 for Windows (GraphPad software, La Jolla, CA). The Wilcoxon signed rank test, Mann-Whitney U test or ANOVA were used to compare groups as appropriate. For correlations the Spearman rank test was used. A *p-*value of <0.05 was considered to be significant.

## References

[1] Gabrilovich D (2004). Mechanisms and functional significance of tumour-induced dendritic-cell defects.. Nat Rev Immunol.

[2] Fainaru O, Almog N, Wing Yung C, Nakai K, Montoya-Zavala M (2009). Tumor growth and angiogenesis are dependent on the presence of immature dendritic cells..

[3] Minkis K, Kavanagh DG, Alter G, Bogunovic D, O'Neill D (2008). Type 2 Bias of T cells expanded from the blood of melanoma patients switched to type 1 by IL-12p70 mRNA-transfected dendritic cells.. Cancer Res.

[4] Xu S, Koski GK, Faries M, Bedrosian I, Mick R (2003). Rapid High Efficiency Sensitization of CD8+ T Cells to Tumor Antigens by Dendritic Cells Leads to Enhanced Functional Avidity and Direct Tumor Recognition Through an IL-12-Dependent Mechanism.. J Immunol.

[5] Della Porta M, Danova M, Rigolin GM, Brugnatelli S, Rovati B (2005). Dendritic cells and vascular endothelial growth factor in colorectal cancer: correlations with clinicobiological findings.. Oncology.

[6] Osada T, Chong G, Tansik R, Hong T, Spector N (2008). The effect of anti-VEGF therapy on immature myeloid cell and dendritic cells in cancer patients.. Cancer Immunol Immunother.

[7] Mimura K, Kono K, Takahashi A, Kawaguchi Y, Fujii H (2007). Vascular endothelial growth factor inhibits the function of human mature dendritic cells mediated by VEGF receptor-2.. Cancer Immunol Immunother.

[8] Gabrilovich DI, Chen HL, Girgis KR, Cunningham HT, Meny GM (1996). Production of vascular endothelial growth factor by human tumors inhibits the functional maturation of dendritic cells.. Nat Med.

[9] Genentech (2009). “Avastin..

[10] Soria G, Ben-Baruch A (2008). The inflammatory chemokines CCL2 and CCL5 in breast cancer.. Cancer Lett.

[11] Baier PK, Eggstein S, Wolff-Vorbeck G, Baumgartner U, Hopt UT (2005). Chemokines in human colorectal carcinoma.. Anticancer Res.

[12] Payne AS, Cornelius LA (2002). The role of chemokines in melanoma tumor growth and metastasis.. J Invest Dermatol.

[13] Xu L, Warren M, Rose W, Gong W, Wang J (1996). Human recombinant monocyte chemotactic protein and other C-C chemokines bind and induce directional migration of dendritic cells in vitro.. J Leukoc Biol.

[14] Walz A, Schmutz P, Mueller C, Schnyder-Candrian S (1997). Regulation and function of the CXC chemokine ENA-78 in monocytes and its role in disease.. J Leukoc Biol.

[15] Bechara C, Chai H, Lin PH, Yao Q, Chen C (2007). Growth related oncogene-alpha (GRO-alpha): roles in atherosclerosis, angiogenesis and other inflammatory conditions.. Med Sci Monit.

[16] Strieter RM, Belperio JA, Phillips RJ, Keane MP (2004). CXC chemokines in angiogenesis of cancer.. Semin Cancer Biol.

[17] Salcedo R, Ponce ML, Young HA, Wasserman K, Ward JM (2000). Human endothelial cells express CCR2 and respond to MCP-1: direct role of MCP-1 in angiogenesis and tumor progression.. Blood.

[18] Sozzani S, Luini W, Borsatti A, Polentarutti N, Zhou D (1997). Receptor expression and responsiveness of human dendritic cells to a defined set of CC and CXC chemokines.. J Immunol.

[19] Feijoo E, Alfaro C, Mazzolini G, Serra P, Penuelas I (2005). Dendritic cells delivered inside human carcinomas are sequestered by interleukin-8.. Int J Cancer.

[20] Gorman S, Tosetto M, Lyng F, Howe O, Sheahan K (2009). Radiation and chemotherapy bystander effects induce early genomic instability events: Telomere shortening and bridge formation coupled with mitochondrial dysfunction.. Mutat Res.

[21] Liu WM, Fowler DW, Smith P, Dalgleish AG (2009). Pre-treatment with chemotherapy can enhance the antigenicity and immunogenicity of tumours by promoting adaptive immune responses.. Br J Cancer.

[22] Alfaro C, Suarez N, Gonzalez A, Solano S, Erro L (2009). Influence of bevacizumab, sunitinib and sorafenib as single agents or in combination on the inhibitory effects of VEGF on human dendritic cell differentiation from monocytes.. Br J Cancer.

[23] Liu C, Lin J, Zhao L, Yang Y, Gao F (2011). Gamma-ray irradiation impairs dendritic cell migration to CCL19 by down-regulation of CCR7 and induction of cell apoptosis.. Int J Biol Sci.

[24] Saraiva M, O'Garra A (2010). The regulation of IL-10 production by immune cells.. Nat Rev Immunol.

[25] KIKUCHI K, KUSAMA K, SANO M, NAKANISHI Y, ISHIGE T (2006). Vascular Endothelial Growth Factor and Dendritic Cells in Human Squamous Cell Carcinoma of the Oral Cavity.. Anticancer Res.

[26] de la Rosa G, Longo N, Rodriguez-Fernandez JL, Puig-Kroger A, Pineda A (2003). Migration of human blood dendritic cells across endothelial cell monolayers: adhesion molecules and chemokines involved in subset-specific transmigration.. J Leukoc Biol.

[27] Bharadwaj U, Li M, Zhang R, Chen C, Yao Q (2007). Elevated Interleukin-6 and G-CSF in Human Pancreatic Cancer Cell Conditioned Medium Suppress Dendritic Cell Differentiation and Activation.. Cancer Res.

[28] Prechtel AT, Steinkasserer A (2007). CD83: an update on functions and prospects of the maturation marker of dendritic cells.. Arch Dermatol Res.

[29] Breloer M, Fleischer B (2008). CD83 regulates lymphocyte maturation, activation and homeostasis.. Trends Immunol.

[30] Czerniecki BJ, Cohen PA, Faries M, Xu S, Roros JG (2001). Diverse functional activity of CD83+ monocyte-derived dendritic cells and the implications for cancer vaccines.. Crit Rev Immunol.

[31] Conti I, Rollins BJ (2004). CCL2 (monocyte chemoattractant protein-1) and cancer.. Semin Cancer Biol.

[32] Kulbe H, Levinson NR, Balkwill F, Wilson JL (2004). The chemokine network in cancer--much more than directing cell movement.. Int J Dev Biol.

[33] Braun MC, Lahey E, Kelsall BL (2000). Selective Suppression of IL-12 Production by Chemoattractants.. J Immunol.

[34] Omata N, Yasutomi M, Yamada A, Iwasaki H, Mayumi M (2002). Monocyte Chemoattractant Protein-1 Selectively Inhibits the Acquisition of CD40 Ligand-Dependent IL-12-Producing Capacity of Monocyte-Derived Dendritic Cells and Modulates Th1 Immune Response.. J Immunol.

[35] Tazzyman S, Lewis CE, Murdoch C (2009). Neutrophils: key mediators of tumour angiogenesis.. Int J Exp Pathol.

[36] Braun MC, Kelsall BL (2001). Regulation of interleukin-12 production byG-protein-coupled receptors.. Microbes Infect.

[37] Puig-Kroger A, Relloso M, Fernandez-Capetillo O, Zubiaga A, Silva A (2001). Extracellular signal-regulated protein kinase signaling pathway negatively regulates the phenotypic and functional maturation of monocyte-derived human dendritic cells.. Blood.

[38] Wilson C, Purcell C, Seaton A, Oladipo O, Maxwell PJ (2008). Chemotherapy-induced CXC-chemokine/CXC-chemokine receptor signaling in metastatic prostate cancer cells confers resistance to oxaliplatin through potentiation of nuclear factor-kappaB transcription and evasion of apoptosis.. J Pharmacol Exp Ther.

[39] Nguyen-Jackson H, Panopoulos AD, Zhang H, Li HS, Watowich SS (2010). STAT3 controls the neutrophil migratory response to CXCR2 ligands by direct activation of G-CSF-induced CXCR2 expression and via modulation of CXCR2 signal transduction.. Blood.

[40] Shyamala V, Khoja H (1998). Interleukin-8 receptors R1 and R2 activate mitogen-activated protein kinases and induce c-fos, independent of Ras and Raf-1 in Chinese hamster ovary cells.. Biochemistry.

